# A 0.01-degree gridded precipitation dataset for Japan, 1926-2020

**DOI:** 10.1038/s41597-022-01548-3

**Published:** 2022-07-19

**Authors:** Misako Hatono, Masashi Kiguchi, Kei Yoshimura, Shinjiro Kanae, Koichiro Kuraji, Taikan Oki

**Affiliations:** 1 Graduate School of Advanced Science and Engineering, Hiroshima University, 1-4-1 Kagamiyama, Higashi-Hiroshima, Hiroshima 739-8527 Japan; 2 Institute for Future Initiatives, The University of Tokyo, 7-3-1 Hongo, Bunkyo, Tokyo 113-8654 Japan; 3 Institute of Industrial Science, The University of Tokyo, 5-1-5 Kashiwanoha, Kashiwa, Chiba 277-8574 Japan; 4 School of Engineering, Tokyo Institute of Technology, 2-12-1 Ookayama, Meguro, Tokyo 152-8550 Japan; 5 Graduate School of Agricultural and Life Sciences, The University of Tokyo, 1-1-1 Yayoi, Bunkyo, Tokyo 113-8657 Japan; 6 Graduate School of Engineering, The University of Tokyo, 7-3-1 Hongo, Bunkyo, Tokyo 113-8656 Japan

**Keywords:** Hydrology, Hydrology, Natural hazards

## Abstract

We developed a 0.01-degree gridded precipitation dataset of Japan based on historical observation datasets covering 1926 to 2020. Historical observations conducted by the Japan Meteorological Agency and other Japanese bureaucratic agencies were spatially interpolated using the inverse distance weighting method at daily and hourly temporal resolutions. Optimal parameterization for our interpolation process was selected by comparing interpolated results of various parameter combinations with precipitation observation conducted by the University of Tokyo Forests. We conducted cross-validation for over 1,000 stations with sufficient data throughout our data period and verified our product can reproduce the temporal variability of local precipitation. The strong points of our precipitation dataset are its high spatiotemporal resolution and the abundance of point precipitation source data. We expect our dataset to be highly relevant to various future studies as it can serve multiple purposes such as forcing data for hydrological models or a database for analyzing the characteristics of historical rainfall events.

## Abstract (summary)

**Table:** 

Measurement(s)	Precipitation
Technology Type(s)	Weather station
Sample Characteristic - Environment	Climate system
Sample Characteristic - Location	Japan

## Background & Summary

Typhoon Prapiroon formed off the coast of Japan on June 29, 2018 and transformed into an extratropical cyclone over the Japan Sea on July 4. The stationary front over western Japan after July 5, combined with the already humid air from the typhoon, continued to supply large amounts of moisture and resulted in heavy rainfall across Japan, especially in the western region^1^. This extreme precipitation event caused several levee breaks and landslides, resulting in 221 deaths, 390 injuries and over 6,000 completely destroyed houses^2^. Fujibe (2018)^3^ used historical point observations from local meteorological observatories from 1901 and indicated that, in this heavy rain event, 12 stations had daily precipitation ranking in the top ten. The Japan Meteorological Agency (hereinafter referred to as JMA) also estimated the return periods for each observation station registered in Automated Meteorological Data Acquisition System (hereinafter referred to as AMeDAS). While these insightful estimations were made using only precipitation observed at certain stations, a detailed spatial distribution of precipitation is crucial when assessing rainfall events in regions of Japan that lack dense observation networks. In addition to analyzing precipitation patterns, gridded precipitation datasets have also been used as input for hydrological modelling and flood analysis in gauged and ungauged basins^4–6^. As many earlier studies have indicated^6–8^, the spatial variability obtained from gridded precipitation datasets can also be crucial when assessing the severity and characteristics of an extreme precipitation event and its subsequent flooding.

There are various gridded datasets based on observations that cover Japan (e.g., GSMaP, RadarAMeDAS, REGEN)^9–12^, but most products either cover relatively short time periods or have coarse resolution. One product that overcomes these limitations is APHRO_JP, which is a 0.05-degree long-term daily precipitation dataset that covers 1900–2008^13^. Since its focus was to develop a high-resolution dataset with consistent quality throughout the dataset period, APHRO_JP uses limited data available from JMA. For the historical period covering 1901–1976, APHRO_JP uses less than 200 stations across Japan, which is insufficient for use in cases of extreme events in very localized areas. Additionally, a 0.05-degree spatial resolution (i.e., approximately 20 km^2^ near Tokyo) can sometimes be too coarse for flood analysis in Japan where the catchment area of the smallest class A river, which are deemed important for national economy, is approximately 130 km^2^.

Here, we utilized all available point observations to develop a highly detailed gridded precipitation dataset. Our dataset was constructed with 0.01-degree spatial resolution (i.e., approximately 0.8 km^2^ near Tokyo) at hourly and daily temporal resolutions depending on the data source. Apart from the improved spatiotemporal resolutions, one of the major improvements of our dataset compared to other gridded datasets is the abundance of data. For the 1926–1975 historical period, we significantly increased the number of stations to over 1000. In order to utilize the large historical precipitation dataset digitized by other researchers that included only the station names, we identified and allocated geographical coordinates for most stations by digitizing metadata listed in various historical reports. For the latter 1976–2020 period, we utilized point observations from the Ministry of Land, Infrastructure, Transport and Tourism (hereinafter referred to as MLIT) in addition to the data from JMA utilized in APHRO_JP, which nearly doubled the number of available point observation sites. Although we anticipate the increase in the source observation to be beneficial, Masson and Frei^14^ shows irregular patterns in long-term trends when using temporally varying station networks. Users should consider this fact to determine whether our dataset is well-suited for their intended purpose.

## Methods

**Fig. 1:**
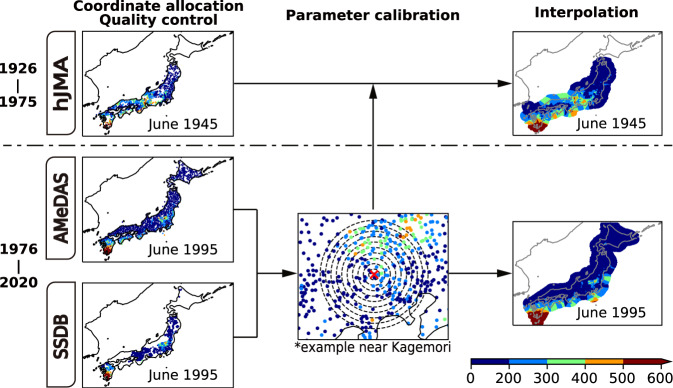
Flow diagram of the method in this study. The color indicates monthly precipitation [mm/mon].

A flow diagram of our method is shown in Fig. 1.

### Observation dataset

Meteorological observations have been conducted by JMA since the beginning of the 1900s. Daily precipitation records since 1926 from over one thousand stations have been digitized from image data stored in CD-ROMs (hereinafter referred to as hJMA). We note that daily precipitation in this dataset was recorded with different starting hours of the day (i.e., 09:00 and 10:00 JST) depending on the year^15^. Because the coordinates of each station were not listed in the original dataset, we utilized various documents to identify the geographical location of each observation station. We also considered certain location changes that happened over time. We encourage readers who are interested in the digitized point precipitation dataset to contact the corresponding researchers for further details.

AMeDAS is a regional meteorological observation system operated by JMA. Various observations of variables such as precipitation and air temperature are conducted automatically and published on their website in real time. A tipping-bucket rain gauge is used for precipitation observation by JMA. AMeDAS started its operation on November 1, 1974, and currently has around 1,300 stations at approximately 17 km intervals. We used hourly data from 1976 to 2020. Most of the full dataset utilized in this study was purchased in CD-ROM format from Japan Meteorological Business Support Center. AMeDAS data are currently available through the JMA website (https://www.data.jma.go.jp/obd/stats/etrn/index.php). We note that the minimum precipitation threshold in the acquired media was 1 mm.

Water Information System (in Japanese: Suimon Suishitsu Database; hereinafter referred to as SSDB) is a database that archives historical observations collected by regional development bureaus of the MLIT. Various observations such as precipitation and water quality have been collected and archived in near real-time. Although data in SSDB is available since the 1930s, we used hourly data from 1976 to 2020 to match that of AMeDAS. Data is available through the SSDB website (http://www1.river.go.jp).

Different quality control (hereinafter referred to as QC) measures are conducted by JMA and MLIT to ensure the published observation dataset do not contain errors. For example, Automatic Quality Control is conducted by JMA for AMeDAS before distribution based on various factors such as historical records exceedance and equipment malfunction^16^. QC by MLIT is also conducted considering similar factors^17^. In addition, we conducted some QC during our interpolation process to exclude missed outliers. For daily precipitation, we excluded values exceeding the historical maximum 24-hour precipitation record of 1,317 mm recorded at Tokushima in 2004^18^. For hourly precipitation, we excluded values exceeding the historical maximum record of 187 mm recorded at Nagasaki in 1982^18^. It should be noted that additional QC is sometimes conducted by JMA and MLIT after data publication. Therefore, some of the observed precipitation values utilized in this study may be different from the most recently available data in each corresponding website. For example, the earlier periods in the AMeDAS dataset tend to have missing values which were later corrected. During the coordinate allocation process for hJMA stations, we manually checked for any errors in the data entry process by plotting all stations for each prefecture on a map and checking for any obvious outliers. If there were any strings in the digitized hJMA precipitation dataset, we edited the value based on information in the metadata and set as invalid if there were no information available.

**Fig. 2:**
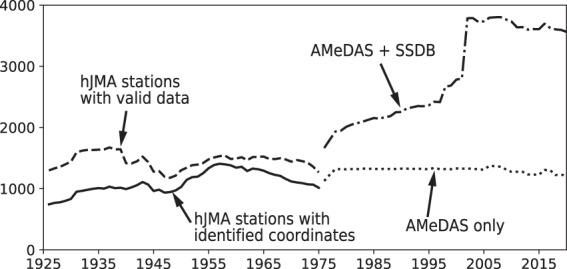
Temporal variation of the number of available observation stations. Solid line indicates the number of hJMA stations for which we were able to identify the coordinates; dashed line indicates the total number of hJMA stations that have valid data; dotted line indicates the number of AMeDAS stations; dash-dotted line indicates the sum of AMeDAS and SSDB stations.

**Fig. 3:**
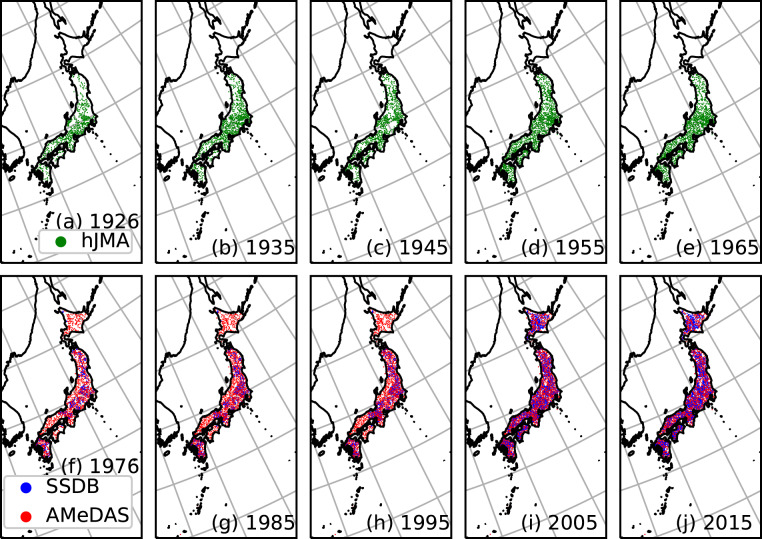
Spatial distribution of the available observation stations. (**a**–**e**) Spatial distribution of available hJMA stations in June of each year. (**f**–**j**) Spatial distribution of available AMeDAS and SSDB stations in June of each year. Red and blue dots represent AMeDAS and SSDB stations, respectively.

Fig. 2 shows the temporal change in the number of stations with at least one valid data entry for each year throughout the data period. The solid line indicates the number of hJMA stations for which we were able to identify the coordinates; the dashed line indicates the total number of hJMA stations with valid data; the dotted line indicates the number of AMeDAS stations; the dash-dotted line indicates the sum of AMeDAS and SSDB stations. Fig. 3(a–e) show the spatial distribution of the hJMA observation stations that were available in 1926, 1935, 1945, 1955 and 1965, respectively. Fig. 3(f–j) show the spatial distribution of AMeDAS and SSDB observation stations in 1976, 1985, 1995, 2005 and 2015, respectively. Stations available in June in each year are shown for simplicity. Apparent regions with no hJMA stations are prefectures for which we were thus far unable to find station coordinates. AMeDAS had similar number of stations compared to hJMA, which is reasonable considering that they are managed by the same agency. The additional SSDB stations enabled a more detailed spatial distribution in our interpolated dataset. Although many SSDB stations initiated precipitation monitoring in the 1950s, we could not obtain data from earlier periods for many SSDB stations via their website. We will continue our efforts to identify the remaining hJMA station coordinates, expand our observation dataset and update our gridded dataset.

### Spatial interpolation

We applied the inverse distance weighting method (hereinafter referred to as IDW) in this study. The precipitation for grid *j*, *P*_*j*_[mm/T], can be estimated as

1 $${P}_{j}=\frac{{\sum }_{i=1}^{sNum}\frac{{p}_{i}}{{d}_{ij}^{k}}}{{\sum }_{i=1}^{sNum}\frac{1}{{d}_{ij}^{k}}}$$

where *p*_*i*_ [mm/T] and *d*_*ij*_[m] are the precipitation and distance of the *i*th closest station to grid *j*, respectively. Unit T represents either daily or hourly intervals, depending on the data source. *k* is a weighting parameter that represents the extent to which distance from the grid is considered. *sNum* represents the number of observation stations used for interpolation. We set a radius of *r* [km] to search for nearby observation stations at each timestep; if there were fewer than *sNum* stations with valid data within the radius, that grid was considered invalid and we set its value to −999. There have been numerous studies related to the impact of utilizing different spatial interpolation methods. In future updates, we hope to include various versions of our dataset considering different spatial interpolation methods. In the Technical Validation section, we have included a preliminary comparison using the angular distance weighting method (hereinafter referred to as ADW) described in New *et al*.^19^.

### Parameter calibration and validation

Earlier studies have investigated the impact of the parameters in Eq. (1). To consider the optimal parameter settings for our dataset, we used observations collected by the University of Tokyo Forests (hereafter referred to as UTF). We used daily precipitation data from 1990 to 1999 for the parameter calibration and from 2000 to 2009 for validation. Data are available through the UTF website (http://www.uf.a.u-tokyo.ac.jp/research_division/data/kishou/index_english.html). We aggregated daily UTF precipitation to monthly precipitation to minimize the impact of different daily boundaries at some stations. For this analysis, we also aggregated our interpolated hourly precipitation data to monthly precipitation. If there were more than two days’ worth of missing data in a month, the respective monthly precipitation value would be deemed invalid and set to −999 to avoid an obvious underestimation. If a station had more than 12 months that were deemed invalid in either of the 10-year periods, it was excluded from our analysis. Four out of fourteen stations with data from 1990 onwards were excluded based on this criteria. *k* in Eq. (1) was set to be between 0 and 5 with 0.5 increments and the number of closest stations *sNum* was set to range between 3 and 15. We also explored the option of using all available stations when there were more than 15 stations within radius *r*, which was adjusted to every 10 km between 10 and 100 km. The total number of parameter combinations considered for all stations was 2,475, and the combination patterns for each station ranged between 220 and 264. The combination patterns differed among the stations depending on the radius necessary to obtain *sNum* stations. For evaluation, we used the Nash-Sutcliffe efficiency (hereafter referred to as *NSE*) which can be calculated as

2 $$NSE=1-\frac{{\sum }_{t=1}^{T}{\left({P}_{m}^{t}-{P}_{o}^{t}\right)}^{2}}{\mathop{\sum }\limits_{t=1}^{T}{\left(\overline{{P}_{o}}-{P}_{o}^{t}\right)}^{2}}$$

where ${P}_{m}^{t}$ is our interpolated monthly precipitation at month *t*, ${P}_{o}^{t}$ is the UTF monthly precipitation at month *t*, and $\overline{{P}_{o}}$ is the UTF average monthly precipitation. *NSE* ranges from -*∞* to 1, where 1 indicates that our interpolated values match perfectly with the UTF precipitation. *NSE* is frequently utilized in hydrological studies because of its ability to evaluate variability and seasonality. Regarding the criteria, Moriasi *et al*.^20^ considered *NSE* s larger than 0.65 and 0.75 as good and very good, respectively. Although these thresholds were set for river discharge, they are still useful for qualitative comparison.

**Fig. 4:**
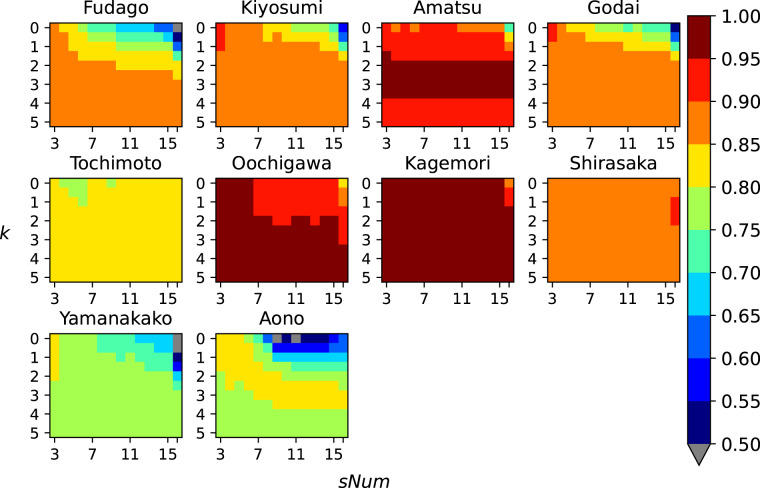
Impact assessment of parameter calibration. Difference in *NSE* due to various *k* and *sNum* combinations.

The average *NSE* in each UTF station had good accuracy ranging between 0.73 and 0.97. Fig. 4 shows the difference in *NSE* based on *sNum* and *k* combinations. If multiple *r* options were available for a given combination, the average *NSE* is shown. Precipitation at stations with relatively low *NSE* has irregular characteristics, as they are located in unique terrains without JMA stations nearby. Overall, *NSE* had relatively small fluctuations, although *NSE* seemed to decrease with combinations of larger *sNum* and smaller *k*. This may be because in order to use a larger *sNum*, the selected observation stations would be located further away from the UTF station. Therefore, larger *k* would be suitable to put more weight on those closer to the target site. This characteristic can also be seen when conducting a two-sample Kolmogorov-Smirnov test, whose null hypothesis is that the two distributions are identical. We examined the impact of *sNum* and *k* with the Kolmogorov-Smirnov test by adjusting one parameter and keeping the other fixed. For example, distributions with small and large *sNum* while keeping *k* fixed at 0 to 1.5 could reject the null hypothesis (i.e., p < 0.1). On the other hand, distributions with small and large *k* while keeping *sNum* fixed at over 10 could reject the null hypothesis. Based on these findings, we deemed it acceptable to use *k* = 2 which has been generally utilized in numerous studies using IDW^21,22^. Because *sNum* did not seem to have a significant impact on the interpolated time-series when *k* is over 1.5, we decided to use *sNum* = 3 to minimize the calculation cost. When considering the optimal search radius for this period, we were able to find at least three valid observation stations within 30 km of each grid containing a UTF site. Because the observation network for the first half of our dataset is relatively sparse, we decided to use *r* = 100 *km* which enabled interpolation of grids on most of the main island of Japan. We will be updating this radius in the future, as we continually find more observations that can be utilized in our dataset. From the parameter calibration results, we estimated the precipitation in each grid as follows:

3 $$\begin{array}{c}{P}_{j}=\frac{{\sum }_{i=1}^{3}\frac{{p}_{i}}{{d}_{ij}^{2}}}{{\sum }_{i=1}^{3}\frac{1}{{d}_{ij}^{2}}}\end{array}$$

The validation using UTF data for 2000 to 2009 is shown in the Technical Validation section.

## Data Records

### UTF data for calibration and validation

To quantitatively compare our data with observations not included in our interpolation process, we used precipitation observations conducted by the UTF. Meteorological observations, including daily precipitation using tipping-bucket rain gauges since 1989, are freely available through their website (http://www.uf.a.u-tokyo.ac.jp/research_division/data/kishou/index_english.html). We decided to aggregate daily values to monthly values because boundary times of daily data were different among sites. We used 1990 to 1999 for parameter calibration and 2000 to 2009 for validation. The coordinates of the stations are listed in Table 1.

**Table 1:** Coordinates of UTF observation sites.

Name	Latitude	Longitude
Fudago	35°12′00″N	140°08′35″E
Kiyosumi	35°09′35″N	140°08′45″E
Amatsu	35°07′35″N	140°09′11″E
Godai	35°11′35″N	140°06′35″E
Tochimoto	35°56′40″N	138°51′47″E
Oochigawa	35°54′32″N	138°58′43″E
Kagemori	35°59′00″N	139°04′36″E
Shirasaka	35°13′08″N	137°19′53″E
Yamanakako	35°24′27″N	138°51′52″E
Aono	34°41′29″N	138°50′19″E

### APHRO_JP

We used APHRO_JP to evaluate the characteristics of our long-term dataset. We only used months where there were less than 2-days’ worth of missing data. Their data, including coarser global versions, are available through their website (https://www.chikyu.ac.jp/precip/english/index.html).

### Final dataset

The final dataset is a 0.01-degree gridded precipitation dataset at daily and hourly time resolutions for 1926 to 1975 and 1976 to 2020, respectively. The dataset is stored in netCDF format, archived at Harvard Dataverse^23^.

## Technical Validation

We validated monthly precipitation at ten UTF sites for 2000 to 2009. Precipitation at the UTF sites were compared to the respective 0.01-degree grids that includes each site. We also included APHRO_JP monthly precipitation data at the same sites in the comparison for reference. Table 2 shows the statistics for all stations. All stations exhibited very good accuracy, with *NSE* ranging between 0.86 and 0.95. Overestimation and underestimation were each evident in about half of the stations; therefore, our interpolated data did not exhibit distinct trends compared to the observations. These discrepancies may be due to the slight differences in the location of the UTF sites and our observation sites. There were no significant differences between our interpolated time-series and APHRO_JP. As preliminary comparison for difference in interpolated values with different interpolation methods, we compared *NSE* values in the ten UTF sites using IDW and ADW. The *NSE* difference between the two methods were relatively small, with a less than 0.01 difference at most stations. Although some stations had better accuracy using ADW, the *NSE* for IDW results are very good, nonetheless. As future works, we hope to expand our dataset to include different versions using various interpolation methods.

**Table 2:** Validation results of UTF stations.

Name	Mean monthly precipitation [mm/mon]			*NSE*	
	UTF	This study	APHRO_JP	This study	APHRO_JP
Fudago	207	175	175	0.88	0.89
Kiyosumi	188	175	170	0.94	0.92
Amatsu	164	169	173	0.95	0.94
Godai	202	175	174	0.91	0.90
Tochimoto	127	135	132	0.92	0.94
Oochigawa	135	136	133	0.88	0.93
Kagemori	113	126	115	0.93	0.96
Shirasaka	148	125	146	0.92	0.95
Yamanakako	219	204	188	0.86	0.84
Aono	188	168	182	0.92	0.93

**Fig. 5:**
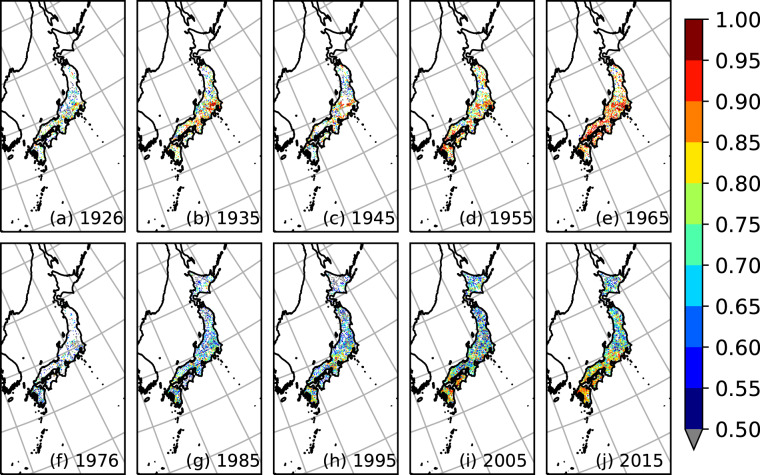
*NSE* at stations included in cross-validaton analysis for each year.

We also conducted cross-validation to investigate the accuracy of our high-resolution dataset. We excluded a certain site from our observation dataset and conducted IDW interpolation for that site using the remaining sites. This was repeated for every observation site. For simplicity and easier data handling, we evaluated the accuracy for each year at stations with less than 30 days’ worth of missing data. On average, 978 and 2298 sites were validated annually in this analysis for 1926–1975 and 1976–2020, respectively. For 1976–2020, we also conducted cross-validation at the daily timescale for comparison. Fig. 5(a–j) shows the spatial distribution of *NSE* at stations included in this analysis. Overall, stations in areas with a dense observation network had especially high accuracy, and more than half of the stations in most years had *NSE* over 0.6. Only the latter period had eight years with a median *NSE* of less than 0.6. There was a significant decrease in *NSE* for hourly precipitation compared to the daily values before 1975, which is most likely because hourly precipitation tends to have larger variations compared to daily values. This can be confirmed with the daily timescale cross-validation for 1976–2020, which had a significant improvement with 0.84 as the smallest median *NSE* in the 45 years. Although our interpolation method is relatively simple compared to other products such as APHRO_JP, the abundance of observation stations seems to provide good accuracy even in higher-resolution grids.

**Fig. 6:**
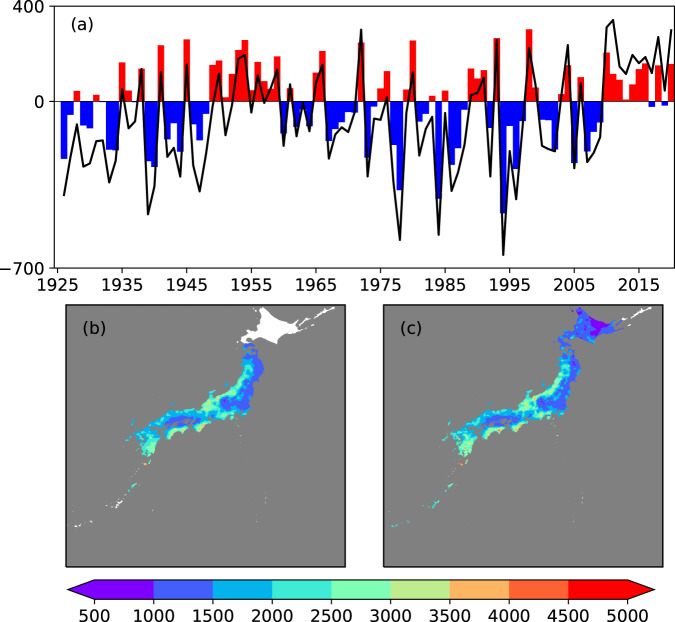
Temporal and spatial distribution of our precipitation dataset. (**a**) Annual precipitation deviation [mm/year] compared to JMA point data averaged over Japan. Hokkaido is excluded in our calculation due to lack of data in earlier periods. Mean annual precipitation [mm/year] of our dataset for the periods (**b**) 1926–1975 and (**c**) 1976–2020. Gray shaded area is not included in the FLOW river network for Japan. White area is where interpolation was not conducted mainly due to lack of observations nearby.

Fig. 6(a) shows the annual precipitation deviation compared with JMA point data. The deviation was calculated following the method described by JMA. First, the deviation at 51 JMA observatories was estimated by comparing each year’s annual total precipitation with the average annual total precipitation of 1991–2020. Subsequently, the annual precipitation deviation for Japan was calculated by averaging the deviation of the 51 stations. We followed this method and applied it to our gridded dataset. Grids with one or more years that had more than 10 days of invalid data were excluded. We also excluded grids that were considered to be oceans in the FLOW river network map^24^. We note that our average values do not include Hokkaido because grids in Hokkaido were mostly deemed invalid in the early period in our dataset. Fig. 6(b,c) show the mean annual precipitation of our dataset for 1926–1975 and 1976–2020, respectively. Although the considered spatial characteristics were different, our results were highly correlated with those of JMA, with R = 0.92.

**Fig. 7:**
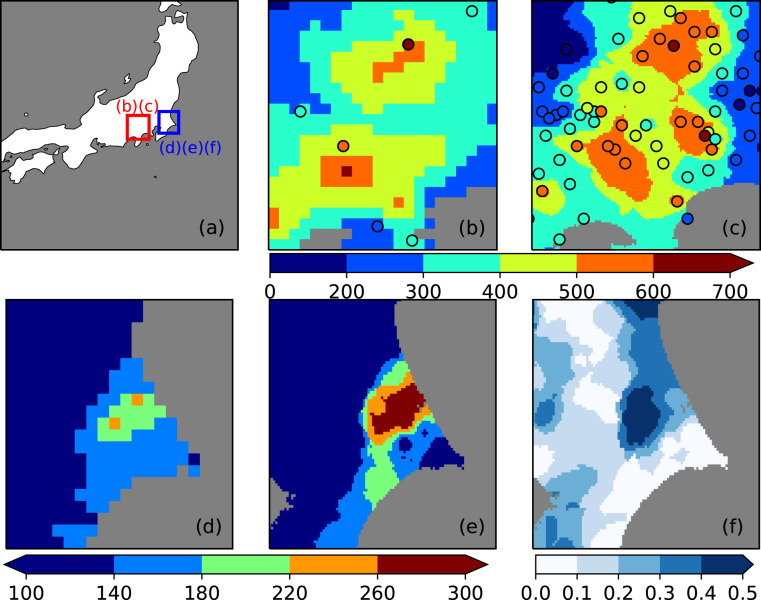
Improvement of spatial and temporal resolution. (**a**) Map of mainland Japan indicating the compared domains. (**b**,**c**) Total precipitation [mm/3dy] for September 13–16, 1947, using APHRO_JP and our dataset, respectively. (**d**,**e**) Daily precipitation [mm/dy] on October 27, 1999, using APHRO_JP and our dataset, respectively. (**f**) Ratio of maximum hourly precipitation to daily precipitation on October 27, 1999 using our dataset. The domains for (**b**,**c**) and (**d**–**f**) are indicated as red and blue squares in map (**a**), respectively.

To confirm the improvements associated with our high temporal and spatial resolutions, we examined two extreme precipitation events in Japan. Typhoon Kathleen brought heavy rainfall in September 1947 and resulted in catastrophic damage in Japan’s largest river basin. At that time, this extreme event was the largest flood since 1910, and is still one of the largest flood events in Japanese history. Fig. 7(b,c) show the total precipitation on September 13–16, 1947 near Mt. Fuji using APHRO_JP and our dataset, respectively. Scatter plots show the total precipitation of the utilized hJMA observations. We note that because we do not have APHRO_JP source data, we plotted for reference the total precipitation of JMA surface observatories in Fig. 7(b), which should be similar to their utilized observation dataset. The domain is shown as a red square in Fig. 7(a). Because of the abundant observations, our dataset is able to exhibit a more detailed spatial distribution. For example, the eastern region, with total rainfall over 500 mm, is not visible in APHRO_JP. In addition, our dataset matches well with observations showing the lower rainfall regions near the east and west boundaries, indicating that the heavy rainfall distribution in this region was narrower than that shown in APHRO_JP. JMA indicates that the maximum hourly precipitation in Japan was 153 mm, which occurred at Katori, Chiba during 19:00–20:00 JST on October 27, 1999. Fig. 7(d–f) show the region around Katori station. The domain is shown as a blue square in Fig. 7(a). Fig. 7(d,e) show the daily precipitation on that day using APHRO_JP and our dataset. Figure 7(f) shows the ratio of hourly precipitation during 19:00–20:00 JST to daily precipitation using our dataset. At Katori station, daily precipitation was 299 mm/dy, indicating that more than half of the daily precipitation occurred in one hour. In Fig. 7(f), hourly precipitation accounts for approximately half of the daily precipitation in many grids. When gridded daily precipitation datasets are used as input in hydrological models, they are usually uniformly distributed to match the input time intervals. In the Katori case, this means that the evenly distributed hourly precipitation would be indicated as only 12 mm/hr which could lead to an underestimation of this short-term heavy rainfall event. With our hourly precipitation, we can consider 24-hour precipitation in addition to daily precipitation, which can increase the amount of precipitation while considering the same time interval. With the improved temporal and spatial resolutions, our dataset will be able to contribute to a better understanding of the characteristics and magnitude of a wide range of precipitation events.

## Usage Notes

Data users should be aware that the precipitation data before 1976 is based on observation data with varying daily boundaries. This should be taken into account if data users aim to compare the early period with daily aggregated precipitation after 1976. Since our main focus was to use as many available observation stations as possible, we did not limit our station to only include homogenized time series. Data users should consider this upon usage for long term analysis. Also, it should be noted the time in this paper and dataset is registered in JST.

## Data Availability

The code used in this study can be accessed alongside the final dataset.
